# Thrombin-mediated vasculogenic mimicry: important lessons to improve anticoagulant therapy of selected cancer patients

**DOI:** 10.1038/s41392-020-00366-6

**Published:** 2020-10-30

**Authors:** C. Arnold Spek

**Affiliations:** 1 Amsterdam UMC, University of Amsterdam, Center of Experimental and Molecular Medicine, Amsterdam, The Netherlands; 2 Amsterdam UMC, University of Amsterdam, Cancer Center Amsterdam, Amsterdam, Netherlands

**Keywords:** Cancer therapy, Lung cancer, Lung cancer

In a recent study published in *Signal Transduction and Targeted Therapy*, Dr. Zhao and colleagues^1^ identify thrombin as a novel mediator of vasculogenic mimicry in non-small-cell lung cancer (NSCLC). This provides important mechanistic insight by which coagulation (factors) may drive tumor progression and several lessons to improve anticoagulant therapy of cancer patients can be learned from the paper.

Vasculogenic mimicry is a process in which tumor cells form fluid-conducting vascular-like structures for the perfusion of rapidly growing tumors. Distinct from classical tumor angiogenesis, vascular channels formed by vasculogenic mimicry are lined by differentiated tumor cells with cancer stem cell characteristics and not by endothelial cells. Vasculogenic mimicry is associated with tumor invasion, metastasis, and poor clinical outcomes based upon which vasculogenic mimicry emerged as a potential novel target for anti-tumor therapy. Dr. Zhao and colleagues^1^ underscore this notion and show that vasculogenic mimicry is associated with the overall survival of NSCLC patients. In addition, the authors show that thrombin expression levels are increased in vasculogenic mimicry positive patients and that thrombin induces vasculogenic mimicry in a thrombin receptor (i.e., protease-activated receptor (PAR)-1)-dependent manner. In combination with the observation that thrombin inhibition limits vasculogenic mimicry and subsequent metastasis in experimental animal models, these data led the authors to conclude that thrombin is a therapeutic target for NSCLC.

Already in the early eighteen hundreds, it has been recognized that cancer could provoke thromboembolic complications and cancer patients are nowadays well-known to be at increased risk for developing venous thrombosis. More interesting with respect to the Zhao paper,^1^ several case-control studies focusing on the prevention of thrombosis in cancer patients suggested that anticoagulant treatment negatively affect the likelihood to succumb to cancer.^2^ This led to the hypothesis that cancer cells exploit a hypercoagulable state to more efficiently metastasize suggesting that anticoagulants could limit cancer progression and prolong the life expectancy of cancer patients. Initial randomized placebo controlled clinical trials confirmed this notion and low molecular weight heparin (LMWH) increased (for instance) the median overall survival of NSCLC patients from 8 to 13 months.^3^ The subsequent failure of several large clinical trials to confirm the survival benefit of LMWH, however, tempered enthusiasm for anticoagulants in cancer treatment and a recent meta-analysis confirms that anticoagulant treatment does not improve the prognosis of lung cancer patients.^4^

**Fig. 1:**
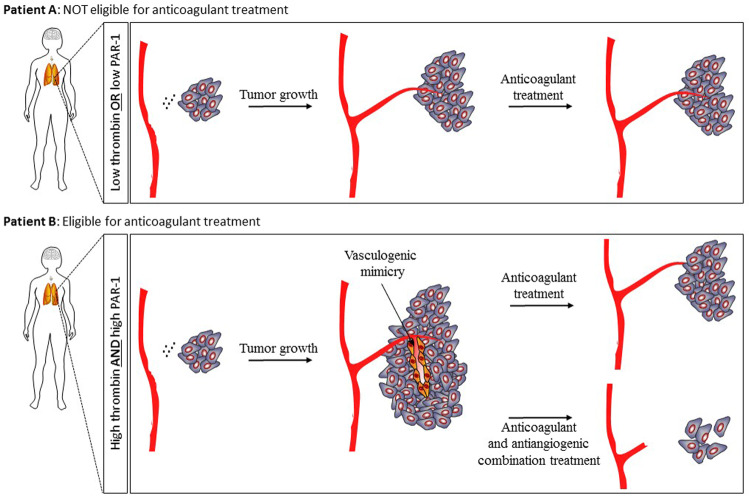
Thrombin-dependent PAR-1 activation induces vasculogenic mimicry thereby driving cancer progression. Patients (like patient A) with low thrombin and/or PAR-1 expression levels, and the consequent lack of vasculogenic mimicry, will not respond to anticoagulant therapy and these patients should be excluded from future anticoagulant trials. Patients (like patient B) with high thrombin and high PAR-1 expression levels, and consequent vasculogenic mimicry, will respond to anticoagulant therapy. The effect of anticoagulants is however limited due to residual growth factor supply through classical angiogenesis and anticoagulants should thus be used in combination with antiangiogenic compounds

The trials based upon which anticoagulants are considered ineffective in (lung) cancer patients did, however, fail to stratify patients on their likelihood to respond to treatment. Especially with anticoagulants, it is pivotal to identify patients likely to benefit from treatment and to exclude probable non-responders. Indeed, anticoagulants induce bleeding complications that may decrease the life expectancy of non-responders thereby masking potential beneficial effects of anticoagulants occurring in a subset of patients. Although no response-predicting biomarkers have yet been characterized, it has been suggested that patients may either be stratified on thrombin or PAR-1 expression levels.^2^ The Zhao paper^1^ however provides compelling evidence that it could actually be the combination of thrombin and PAR-1 expression levels that should be used to stratify patients. Both thrombin and PAR-1 expression levels do, by themselves, not correlate with vasculogenic mimicry and/or overall survival of NSCLC patients. Instead, high PAR-1 levels in combination with thrombin expression in the tumor seems associated with increased vasculogenic mimicry and reduced overall survival. Anticoagulant-dependent thrombin inhibition is thus likely to be especially beneficial in NSCLC patients with thrombin-positive/PAR-1 high tumors and patients without these characteristics should be excluded from future anticoagulant trials (Fig. 1).

Despite the importance of patient stratification for anticoagulant treatment, the identification of thrombin-induced vasculogenic mimicry as an underlying mechanism by which the coagulation system drives NSCLC progression may have more far-reaching implications. Although targeting vasculogenic mimicry may limit the supply of nutrients and oxygen to the tumor, anticoagulants may not be very effective due to the presence of normal blood vessels that still provide tumor cells with essential growth factors. Such a scenario is in perfect agreement with the notion that classic antiangiogenic treatments, that target normal vessels, are of limited clinical use due to residual growth factor supply through vasculogenic mimicry.^5^ Instead of using anticoagulants as single agents, combination therapy with classic antiangiogenic compounds may therefore be preferred (Fig. 1). At least in experimental animals this seem to be the case as thrombin inhibition in combination with the angiogenesis inhibitor gefitinib reduced both tumor growth and spontaneous metastasis more efficient as single agent treatment.^1^

Vasculogenic mimicry is not specific for NSCLC and it thus tempting to speculate that combination therapy may also increase the efficacy of anticoagulants in other cancer types. Although these are exciting thoughts, we have to await properly designed clinical trials to assess whether combination therapy of anticoagulants with angiogenesis inhibitors lives up to its promise. The poor clinical translation of experimental animal experiments in the past makes one humble, however, and tempers enthusiasm.
